# Supplementary material for: NMR-Based Structural Characterization of a Two-Disulfide-Bonded Analogue of the FXIIIa Inhibitor Tridegin: New Insights into Structure–Activity Relationships
Source: Int J Mol Sci. 2021 Jan 17;22(2):880. doi: 10.3390/ijms22020880 (PMC7830451; doi:10.3390/ijms22020880)
Supplement: Supplementary file 1 [file ijms-22-00880-s001.zip › ijms-1068812 suppl for proof/Imhof_IJMS_Special_Issue_Muszbek_FXIIIa_Supp_Info_Resubmission.docx]

Supporting Informations

NMR-based structural characterization of a 2-disulfide bonded analog of the FXIIIa inhibitor tridegin: New insights into structure-activity relationships

Thomas Schmitz ^1^, Ajay Abisheck Paul George ^1,2^, Britta Nubbemeyer ^1^, Charlotte A. Bäuml ^1^, Torsten Steinmetzer ^3^, Oliver Ohlenschläger ^5^, Arijit Biswas ^6^ and Diana Imhof ^1,^*

^1^ Pharmaceutical Biochemistry and Bioanalytics, Pharmaceutical Institute, University of Bonn, An der Immenburg 4, D-53121 Bonn, Germany; t.schmitz@uni-bonn.de (Th.S.); Ajay.PaulGeorge@BioSolveIT.de (A.A.P.G); charlotte.baeuml@uni-bonn.de (C.A.B.); Britta.Nubbemeyer@uni-bonn.de (B.N.);

^2^ BioSolveIT GmbH, An der Ziegelei 79, D-53757 Sankt Augustin, Germany

^3^ Institute of Pharmaceutical Chemistry, Philipps University of Marburg, Marbacher Weg 6, 35032 Marburg, Germany; steinmetzer@uni-marburg.de (To.S.)

^5^ Leibniz Institute on Aging – Fritz-Lipmann-Institute, Beutenbergstr. 11, D-07745 Jena, Germany; Oliver.Ohlenschlaeger@leibniz-fli.de (O.O.)

^6^ Institute of Experimental Hematology and Transfusion Medicine, University Hospital Bonn, Sigmund-Freud-Str. 25, D-53127 Bonn, Germany; arijit.biswas@ukbonn.de (A.B.)

***** Correspondence: dimhof@uni-bonn.de; Tel.: +49-(0)228-735254

Supporting information contains tables, figures and references

Table of contents:

7 tables (Table S1-S7)

6 figures (Figure S1-S6)

**Table S1.** Analytical characterization of the different tridegin peptides.

| **Peptide** | **t_R_ (C18) [min]** | **t_R_ (C8) [min]** | **[M]^calc^**  **[mono]** | **[M]^meas^**  **[M+H]^+^** | **NMR**  **(MHz)** |
| --- | --- | --- | --- | --- | --- |
| **Isomer B_[C19S,C25S]_** | 18.5^a^ | 19.0^a^ | 7745.9 | 7746.9 | 700 |
| **N-terminal part** | 15.7^a^ | 16.3^a^ | 4226.0 | 4227.1 | 700 |
| **C-terminal part** | 13.7^a^ | 14.2^a^ | 3531.9 | 3532.8 | 700 |

^calc^calculated monoisotopic mass; ^meas^ measured monoisotopic mass; ^a^ gradient: 20-60% MeCN (0.1% TFA) in water (0.1% TFA) in 40 min

**Table S2.** Determination of disulfide connectivity by chymotryptic digest of the **N-terminal fragment** and subsequent MS analysis.

| **Disulfide bond** | **Peptide fragment** | **[M]^calc^**  **[mono]** | **[M]^meas^**  **[M+H]^+^** |
| --- | --- | --- | --- |
| C05-C37 | KLLPC_05_KEW-IPQC_37_ | 1471.8 | 1472.8 |
| C17-C31 | C_17_W-C_31_AF | 644.2 | 645.2 |

**Table S3.** NMR resonance assignments for the **N-terminal** and **C-terminal fragment** of isomer B_[C19S,C25S]_.

**C-terminal fragment**

|  |  | **NH** | **Hα** | **Hβ** | **Hγ** | **Hδ** | **Hε** | **Hζ** |
| --- | --- | --- | --- | --- | --- | --- | --- | --- |
| **1** | **Arg** | - | - | 1.87 | 1.65 | - | - | - |
| **2** | **Pro** | - | 4.32 | - | - | - | - | - |
| **3** | **Arg** | 8.41 | 4.18 | 1.72, 1.77 | 1.60, 1.61 | - | - | - |
| **4** | **Ser** | 8.27 | 4.31 | 3.76, 3.81 | - | - | - | - |
| **5** | **Glu** | 8.39 | 4.24 | 1.83, 2.00 | 2.16, 2.20 | - | - | - |
| **6** | **Leu** | 8.06 | 4.25 | 1.51, 1.56 | 1.52 | 0.79, 0.84 | - | - |
| **7** | **Iso** | 7.92 | 4.06 | 1.75 | 0.79, 1.10, 1.38 | 0.76 | - | - |
| **8** | **Lys** | 8.28 | 4.54 | 1.64, 1.74 | 1.37 | 1.62 | 2.93 | - |
| **9** | **Pro** | - | 4.33 | - | - | 3.57, 3.75 | - | - |
| **10** | **Met** | 8.36 | 4.37 | 1.94, 2.02 | 2.49, 2.55 | - | 2.02 | - |
| **11** | **Asp** | 8.18 | 4.51 | 2.56, 2.61 | - | - | - | - |
| **12** | **Asp** | 8.17 | 4.50 | 2.56, 2.63 | - | - | - | - |
| **13** | **Iso** | 7.84 | 3.97 | 1.71 | 0.66, 0.97, 1.04 | 0.70 |  | - |
| **14** | **Tyr** | 8.03 | 4.46 | 2.88, 3.00 | - | 7.06 | 6.74 | - |
| **15** | **Gln** | 7.93 | 4.20 | 1.84, 1.96 | 2.21 | - | 6.74, 7.46 | - |
| **16** | **Arg** | 8.12 | 4.45 | 1.65, 1.74 | 1.59, 1.60 | 3.12 | 7.16 | - |
| **17** | **Pro** |  | 4.36 | 1.79, 2.20 | 1.94 | 3.54, 3.75 | - | - |
| **18** | **Val** | 8.09 | 3.91 | 1.91 | 0.75, 0.83 | - | - | - |
| **19** | **Glu** | 8.21 | 4.14 | 1.73, 1.78 | 1.96, 2.07 | - | - | - |
| **20** | **Phe** | 8.20 | 4.81 | 2.85, 3.08 | - | 7.22 | 7.26 | - |
| **21** | **Pro** | - | 4.32 | 1.82, 2.17 | 1.91 | 3.51, 3.63 | - | - |
| **22** | **Asn** | 8.37 | 4.61 | 2.65, 2.75 | - | 6.84, 7.52 | - | - |
| **23** | **Leu** | 7.97 | 4.56 | 1.50, 1.54 | 1.56 | 0.83, 0.85 | - | - |
| **24** | **Pro** | - | 4.33 | 1.80, 2.19 | 1.91, 1.94 | 3.56, 3.74 | - | - |
| **25** | **Leu** | 8.18 | 4.22 | 1.47, 1.55 | - | 0.80, 0.85 | - | - |
| **26** | **Lys** | 8.18 | 4.53 | 1.63, 1.73 | 1.38 | 1.63 | 2.92 | - |
| **27** | **Pro** | - | 4.44 | 1.84, 2.29 | 1.93, 1.97 | 3.51, 3.68 | - | - |
| **28** | **Arg** | 8.55 | 4.19 | 1.75, 1.80 | 1.61 | 3.15 | 7.16 | - |
| **29** | **Glu** | 8.35 | 4.18 | 1.87, 1.98 | 2.19 | - | - | - |

**N-terminal fragment**

|  |  | **NH** | **Hα** | **Hβ** | **Hγ** | **Hδ** | **Hε** | **Hζ** |
| --- | --- | --- | --- | --- | --- | --- | --- | --- |
| **1** | **Lys** | - | - | - | - | - | - | - |
| **2** | **Leu** | 8.52 | 4.28 | 1.44 | 1.39 | 0.70, 0.74 | - | - |
| **3** | **Leu** | 8.35 | 4.50 | 1.41, 1.46 | 1.52 | 0.75, 0.76 | - | - |
| **4** | **Pro** | - | 4.27 | 1.75, 2.09 | 1.84 | 3.48, 3.65 | - | - |
| **5** | **Cys** | 8.19 | 4.45 | 2.77, 2.97 | - | - | - | - |
| **6** | **Lys** | 8.45 | 4.14 | 1.59 | 1.21 | 1.48 | 2.77 | - |
| **7** | **Glu** | 8.15 | 4.15 | 1.74, 1.87 | 2.07 | - | - | - |
| **8** | **Trp** | 8.07 | 4.39 | 2.98, 3.05 | - | 6.99 | 7.33, 9.92 | 6.93, 7.29, 7.03 (Hη) |
| **9** | **His** | 7.86 | 4.29 | 2.80, 2.93 | - | 6.87 | - | - |
| **10** | **Gln** | 8.03 | 3.93 | 1.77, 1.90 | 2.12 | - | 6.74, 7.38 | - |
| **11** | **Gly** | 8.32 | 3.72, 3.83 | - | - | - | - | - |
| **12** | **Iso** | 7.70 | 4.32 | 1.68 | 0.77, 0.91, 1.24 | 0.64 | - | - |
| **13** | **Pro** | - | - | 1.70, 1.97 | 1.80 | 3.45, 3.64 | - | - |
| **14** | **Asn** | 8.00 | - | 2.52, 2.69 | - | 6.80, 7.48 | - | - |
| **15** | **Pro** | - | 4.02 | 1.72, 1.98 | 1.75 | 3.47, 3.58 | - | - |
| **16** | **Arg** | 8.09 | 4.08 | 1.54, 1.62 | 1.39, 1.44 | 2.91, 2.94 | 7.10 | - |
| **17** | **Cys** | 7.94 | 4.53 | 2.73, 2.88 | - | - | - | - |
| **18** | **Trp** | 7.93 | 4.66 | 3.09, 3.17 | - | 7.06 | 7.41, 9.99 | 6.93, 7.27, 7.02 (Hη) |
| **19** | **Ser** | 8.18 | 4.25 | 3.60, 3.69 | - | - | - | - |
| **20** | **Gly** | 7.70 | 3.60, 3.70 | - | - | - | - | - |
| **21** | **Ala** | 7.87 | 4.12 | 1.19 | - | - | - | - |
| **22** | **Asp** | 8.15 | 4.43 | 2.50, 2.60 | - | - | - | - |
| **23** | **Leu** | 8.03 | 4.09 | 1.49 | 1.44 | 0.67, 0.72 | - | - |
| **24** | **Glu** | 8.28 | 4.06 | 1.83, 1.94 | 2.10, 2.13 | - | - | - |
| **25** | **Ser** | 7.93 | 4.25 | 3.70, 3.74 | - | - | - | - |
| **26** | **Ala** | 8.06 | 4.14 | 1.24 | - | - | - | - |
| **27** | **Gln** | 8.07 | 4.10 | 1.82, 1.96 | 2.18 | 6.67, 7.35 | - | - |
| **28** | **Asp** | 8.04 | 4.39 | 2.53 | - | - | - | - |
| **29** | **Gln** | 8.13 | 4.04 | 1.76, 1.83 | 1.98 | - | 6.67, 7.27 | - |
| **30** | **Tyr** | 8.10 | 4.38 | 2.81, 2.89 | - | 6.93 | 6.63 | - |
| **31** | **Cys** | 8.00 | 4.43 | 2.75, 2.91 | - | - | - | - |
| **32** | **Ala** | 7.93 | 4.08 | 1.13 | - | - | - | - |
| **33** | **Phe** | 7.82 | 4.45 | 2.82, 2.92 | - | 7.01 | 7.13 | 7.10 |
| **34** | **Iso** | 7.70 | 4.19 | 1.63 | 0.70, 0.92, 1.28 | 0.64 | - | - |
| **35** | **Pro** |  | 4.15 | 1.73, 2.07 | 1.80 | 3.43, 3.51 | - | - |
| **36** | **Gln** | 8.32 | 4.20 | 1.78, 1.90 | 2.19 | - | 6.70, 7.34 | - |
| **37** | **Cys** | 8.22 | 4.49 | 2.70, 3.01 | - | - | - | - |

**Table S4.** Energy and structural statistics of the NMR ensembles of the different peptides (lowest energy structures).

|  | **N-terminal fragment** | **C-terminal fragment** | **Isomer B_[C19S,C25S]_** |
| --- | --- | --- | --- |
| total distance restraints   - intra (\|i-j\|=0) - sequential (\|i-j\|=1) - medium (1<\|i-j\|<5) - long range (\|i-j\|≥5) | 171  194  151  48 | 46  54  4  0 | 217  248  155  48 |
| H-bonds (upper + lower) | 14 + 14 | 0 + 0 | 14 + 14 |
| disulfide (upper + lower) | 6 + 6 | 0 + 0 | 6 + 6 |
| angle constraints  (# defined angles) | 378 (54) | 238 (34) | 626 (98) |
| Target function (st. dev.) | 1.92 (0.20) Å^2^ | 0.05 (0.02) Å^2^ | 2.77 (0.31) Å^2^ |
| AMBER physical energies* | -1601.51 kJ/mol | -3155.76 kJ/mol | -5131.18 kJ/mol |
| R.M.S.D.   - heavy atoms (st. dev.) - backbone (st. dev.) | residue 5-37  1.72 (0.40) Å  1.20 (0.39) Å | residue 1-29  9.78 (2.71) Å  8.54 (2.83) Å | residue 5-37  2.12 (0.45) Å  1.51 (0.52) Å |

st. dev. = standard deviation; *Energies were calculated after energy minimization was done in Yasara 19.9.17 using a combination of steepest descents and simulated annealing protocol available in the application with Amber-ff14sb force field.

**Table S5.** Atomic interactions between the docking pose of isomer B_[C19S,C25S]_, which is located at the active site of FXIIIa and FXIIIa. The tryptophans which are part of the hydrophobic tunnel are depicted in red.

| **Interactions** | **FXIIIa amino acid residue** | **Isomer B_[C19S,C25S]_ amino acid residue** |
| --- | --- | --- |
| hydrophobic interactions | Tyr214 | Leu60 |
| (within 5 Å) | Trp279 | Pro13, Pro54, Phe57 |
|  | Ile282 | Trp18 |
|  | Trp370 | Val55, Phe57, Pro58 |
|  | Tyr372 | Pro58, Leu60 |
| hydrogen bonds | Trp279 | Pro54, Glu56, Asn59 |
|  | Asn281 | Asn59 |
|  | Ser290 | Asn59 |
|  | Gln313 | Phe57, Asn59 |
|  | Pro399 | Arg53, Glu56 |
| π-π-interactions  (within 4.5 and 7 Å) | Trp370 | Phe57 |
| cation-π-interaction | Trp279 | Arg53 |
| (within 6 Å) | Trp370 | Arg16 |
|  | Tyr407 | Arg53 |

**Table S6.** Hydrogen bond interactions between the docking pose of isomer B_[C19S,C25S]_, which is located at the active site of FXIIIa and FXIIIa.

| **Pos.^1^** | **Res.^1^** | **Atom^1^** | **Pos.^2^** | **Res.^2^** | **Atom^2^** | **MO** | **Dd-a** | **Dh-a** | **A(d-H-N)** | **A(a-O=C)** |
| --- | --- | --- | --- | --- | --- | --- | --- | --- | --- | --- |
| 279 | TRP | NE1 | 54 | PRO | O | - | 3.00 | 2.59 | 108.11 | 125.27 |
| 313 | GLN | OE1 | 57 | PHE | O | 1 | 3.20 | 2.37 | 132.90 | 118.98 |
| 313 | GLN | OE1 | 57 | PHE | O | 2 | 3.20 | 4.10 | 26.93 | 118.98 |
| 313 | GLN | NE2 | 57 | PHE | O | 1 | 2.85 | 1.88 | 152.48 | 160.82 |
| 313 | GLN | NE2 | 57 | PHE | O | 2 | 2.85 | 3.61 | 37.26 | 160.82 |
| 399 | PRO | O | 53 | ARG | NH1 | 1 | 3.23 | 3.36 | 73.87 | 116.65 |
| 399 | PRO | O | 53 | ARG | NH1 | 2 | 3.23 | 2.64 | 117.15 | 116.65 |
| 399 | PRO | O | 56 | GLU | OE1 | 1 | 2.88 | 2.40 | 106.14 | 135.55 |
| 399 | PRO | O | 56 | GLU | OE1 | 2 | 2.88 | 2.80 | 83.25 | 135.55 |
| 313 | GLN | OE1 | 59 | ASN | N | - | 2.98 | 2.21 | 134.20 | 96.19 |
| 313 | GLN | NE2 | 59 | ASN | N | - | 3.44 | 3.16 | 97.90 | 75.04 |
| 279 | TRP | NE1 | 56 | GLU | OE1 | - | 3.45 | 2.67 | 145.07 | undefined |
| 281 | ASN | OD1 | 59 | ASN | OD1 | 1 | 3.09 | 2.17 | 143.74 | undefined |
| 281 | ASN | OD1 | 59 | ASN | OD1 | 2 | 3.09 | 3.93 | 32.67 | undefined |
| 281 | ASN | ND2 | 59 | ASN | OD1 | 1 | 3.07 | 2.14 | 146.83 | undefined |
| 281 | ASN | ND2 | 59 | ASN | OD1 | 2 | 3.07 | 3.90 | 31.55 | undefined |
| 290 | SER | OG | 59 | ASN | ND2 | - | 3.19 | 9.99 | undefined | undefined |
| 281 | ASN | OD1 | 59 | ASN | OD1 | 1 | 3.09 | 3.34 | 67.78 | undefined |
| 281 | ASN | OD1 | 59 | ASN | OD1 | 2 | 3.09 | 3.00 | 85.25 | undefined |
| 281 | ASN | ND2 | 59 | ASN | OD1 | 1 | 3.07 | 2.84 | 92.21 | undefined |
| 281 | ASN | ND2 | 59 | ASN | OD1 | 2 | 3.07 | 2.72 | 99.45 | undefined |
| 290 | SER | OG | 59 | ASN | ND2 | 1 | 3.19 | 3.11 | 84.53 | undefined |
| 290 | SER | OG | 59 | ASN | ND2 | 2 | 3.19 | 2.50 | 123.57 | undefined |

^1^ belongs to FXIIIa; ^2^ belongs to isomer B_[C19S,C25S]_; Pos.= position; Res.= residue; MO= multiple occupancy; Dd-a= distance between donor and acceptor; Dh-a= distance between hydrogen and acceptor; A(d-H-N)= angle between donor-H-N; A(a-O=C)= angle between acceptor-O=C.

**Table S7.** π-π interactions and cation-π interactions between the docking pose of isomer B_[C19S,C25S]_, which is located at the active site of FXIIIa and FXIIIa.

| **Pos.^1^** | **Res.^1^** | **Pos.^2^** | **Res.^2^** | **Distance** | **Dihedral Angle** |
| --- | --- | --- | --- | --- | --- |
| 279 | TRP | 53 | ARG | 3.13 | 100.95 |
| 370 | TRP | 16 | ARG | 5.10 | 74.59 |
| 370 | TRP | 57 | PHE | 6.03 | 149.41 |
| 407 | TYR | 53 | ARG | 5.04 | 32.40 |

^1^ belongs to FXIIIa; ^2^ belongs to isomer B_[C19S,C25S]_; Pos.= position; Res.= residue.


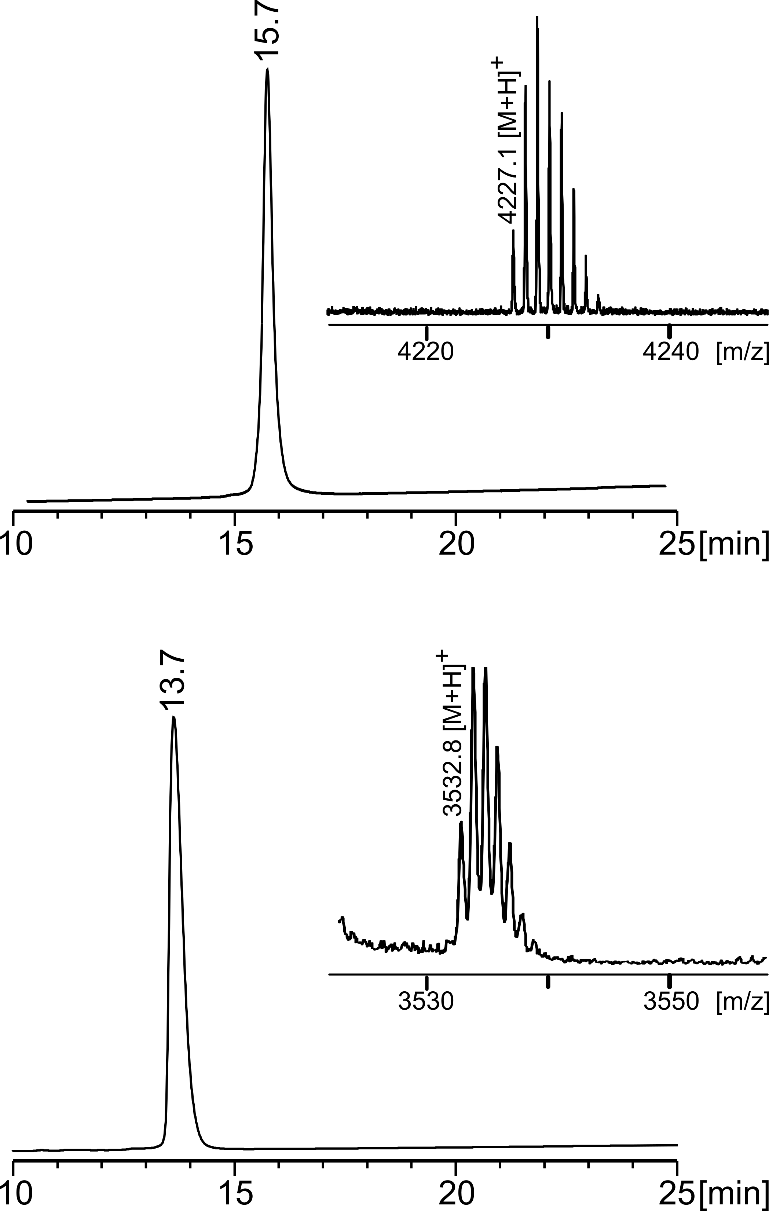


**Figure S1:** Analytical data of the N-terminal (**a**) and C-terminal fragment (**b**). C18 HPLC profile (bottom left) at a gradient of 20-60% eluent B (0.1% TFA in MeCN) in 40 min (eluent A: 0.1% TFA in water) as well as respective MALDI_TOF mass spectra (top left).

**a)**

**b)**


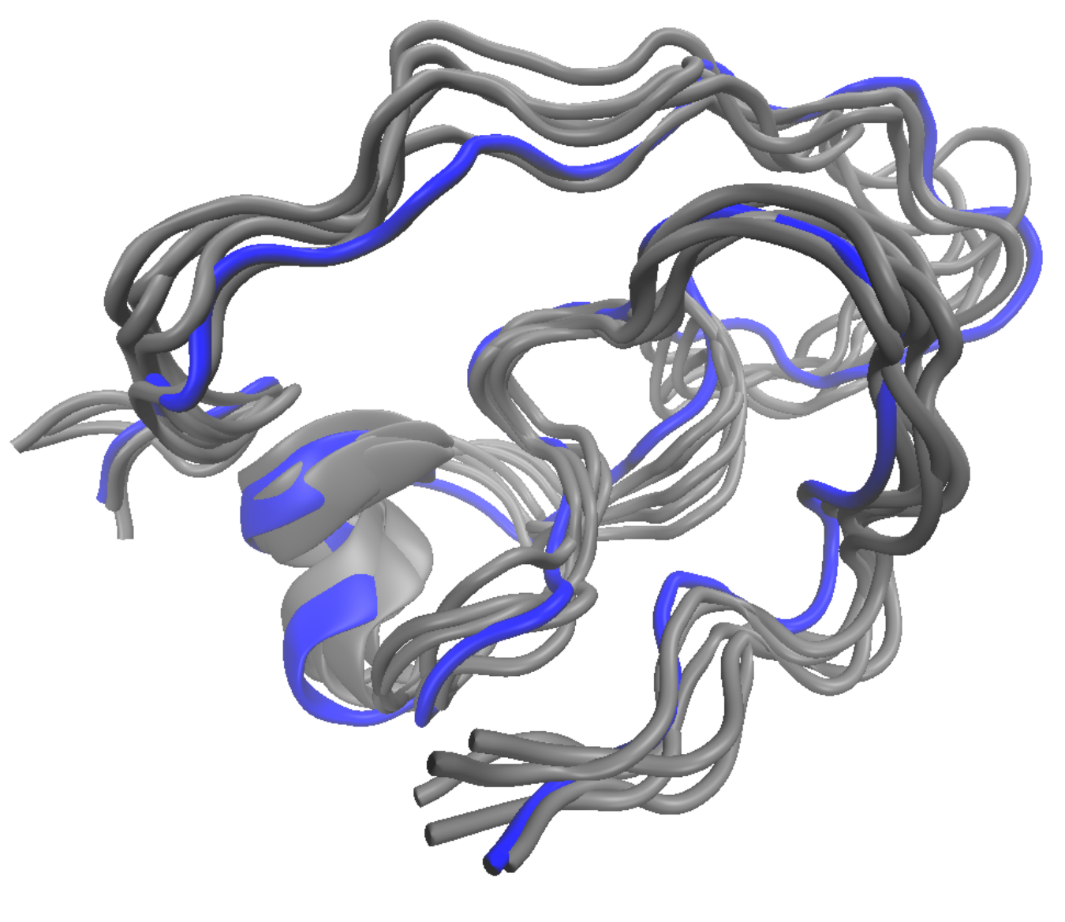


**Figure S2**: Five equidistant structures between 800 and 1000 ns (gray) of a second independent 1000 ns MD simulation superimposed on the final structure from the 300 ns MD simulation (blue). The structures from the second independent simulation aligns very well with the structure from the 300 ns simulation confirming that the structure at 300 ns is valid to be used for docking and further analyses.


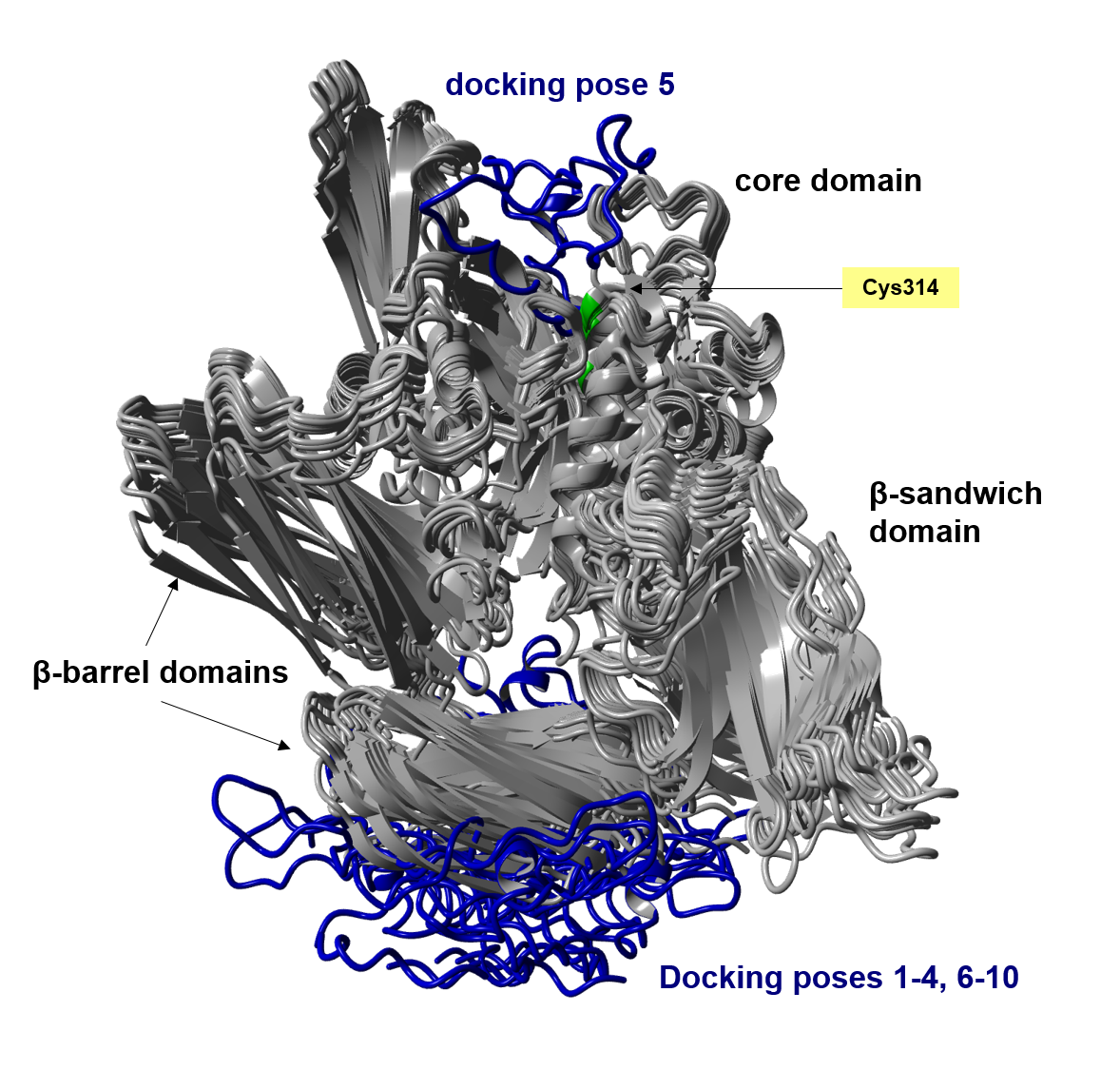


**Figure S3:** Docking of the isomer B_[C19S,C25S]_ model with FXIIIa. The top ten docking poses (blue) are docked on the structure of FXIIIa (grey). Most of the docks are clustered at one of the β-barrel domains (docking pose 1-4,6-10), whereas one of the docking poses (docking pose 5) binds directly next to the catalytic site of FXIIIa.


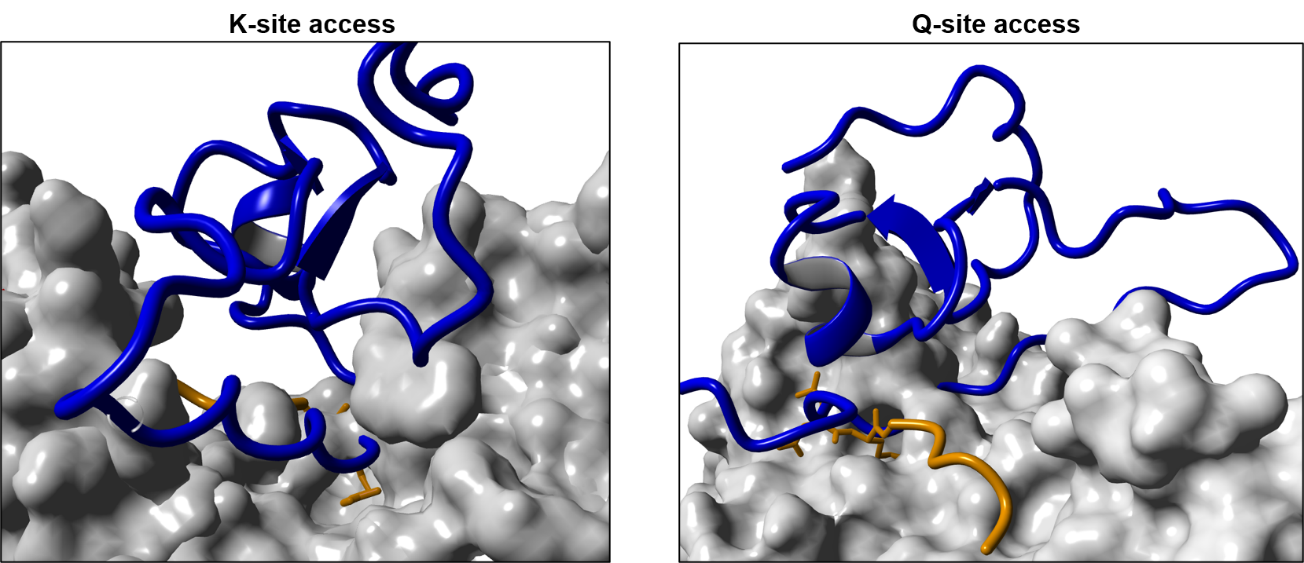


**Figure S4:** Comparison between the inhibition modes of isomer B_[C19S,C25S]_ (blue) and ZED1301 (bronze) to FXIIIa. Zoom-in to the active site of FXIIIa (grey) from to different directions. Left: K-site access shows the region where normally the FXIIIa substrate lysine binds to the active site. Right: Q-site access shows the active site from the opposite of the hydrophobic tunnel where the substrate glutamine interacts with FXIIIa. Both inhibitors occupy the hydrophobic tunnel and blocks the catalytic site (Cys314) for substrates.


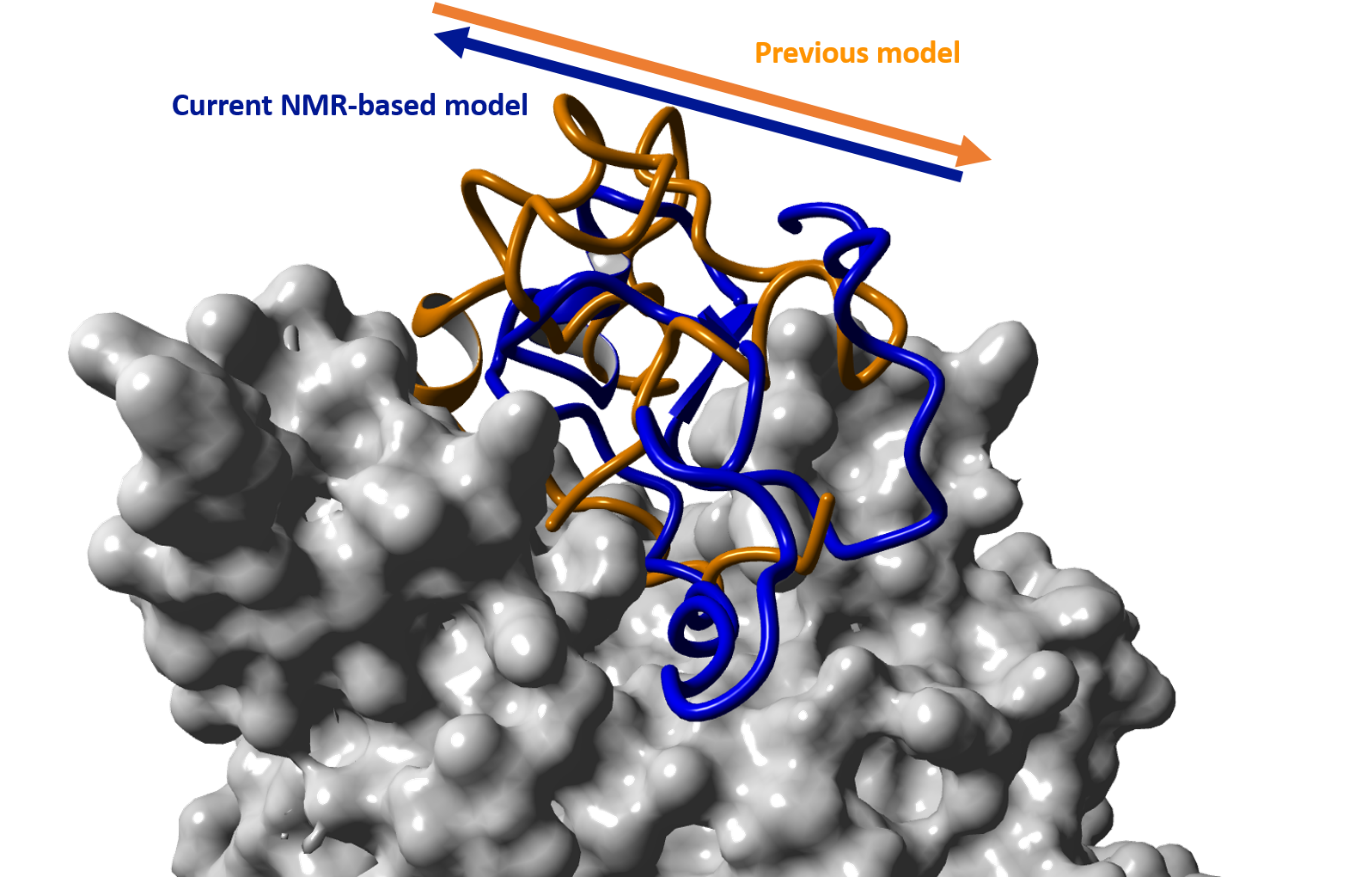


**Figure S5:** Comparison between the inhibition modes of the NMR-based (blue) and the previously reported structure (bronze) of isomer B_[C19S,C25S]_ to FXIIIa. Both inhibitors occupy the hydrophobic tunnel and blocks the catalytic site (Cys314) for substrates. The arrows represent the directions the C-terminal parts of the different structures guide through the hydrophobic tunnel of FXIIIa.


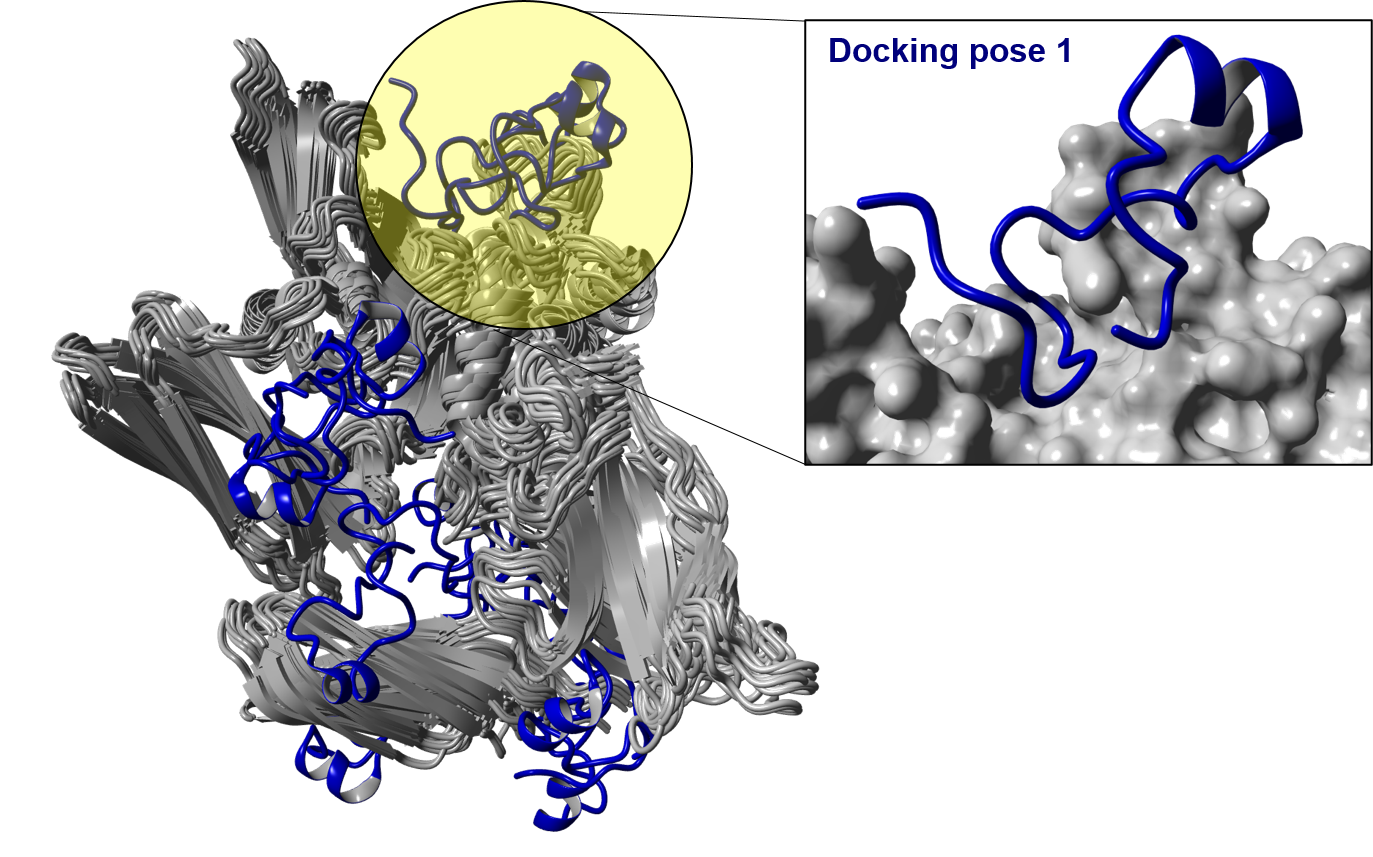


**Figure S6:** Docking of the N-terminal fragment of isomer B_[C19S,C25S]_ with FXIIIa. The top ten docking poses (blue) are docked on the structure of FXIIIa (grey). The best docking pose 1 of the top ten docking poses binds near to the catalytic site of FXIIIa (yellow circle) and is also shown on the zoom-in on the right site.

**Supporting Video**

The video shows the 300 ns MD simulation of lowest energy structure from the 100-member NMR ensemble of the isomer B_[C19S,C25S]._ The NMR structure used as the input for the simulation is shown as a transparent cartoon throught the simulation as reference, while the simulated structure is shown in a red to blue color gradient over the 300 ns time course of the simulation. The secondary structures dynamically updated during the simulation are done via the STRIDE [1] algorithm implemented in VMD 1.9.3 [2].

**Reference**

1. Frishman, D.; Argos, P. Knowledge-based protein secondary structure assignment. *Proteins* **1995**, *23*, 566–579, doi:10.1002/prot.340230412.

2. Humphrey, W.; Dalke, A.; Schulten, K. VMD: Visual molecular dynamics. *Journal of Molecular Graphics* **1996**, *14*, 33–38, doi:10.1016/0263-7855(96)00018-5.
